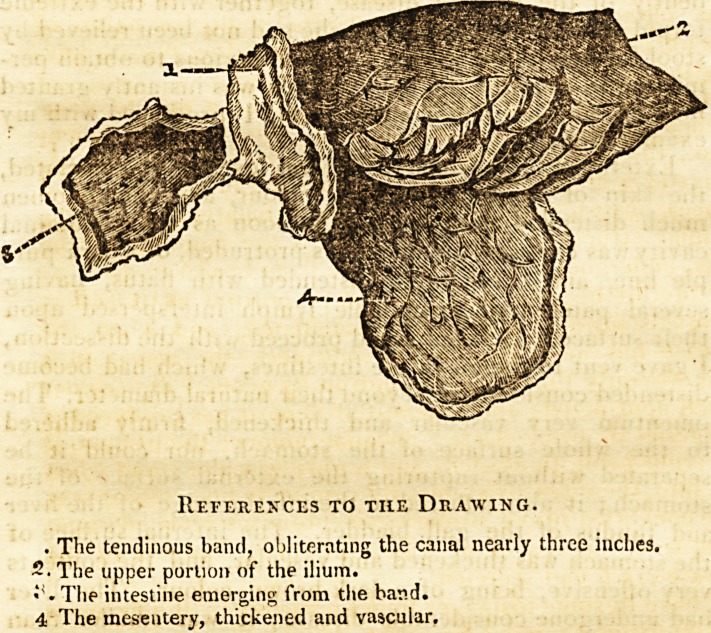# Mr. Bailey's Case of Contracted Intestine

**Published:** 1816-02

**Authors:** H. W. Bailey

**Affiliations:** Thetford


*To the Editors of the Medico-Chirurgical Journal & Review.*

GENTLEMEN,

A Few days since, I was requested to visit Mrs. Goult, aged 72, a pauper belonging to the parish of West Wretham, whom I found expiring as I entered the room. The countenance and whole body were of an extreme yellow colour. From what I could glean from her attendants, I learnt she had been ill six months. She was naturally of a costive habit, as was the case with the rest of her family, all of whom died from the effects of jaundice. I understood she had not been attended by any medical practitioner, and had only taken medicines of an aperient kind, given by her neighbours. At the commencement of her complaint, she was seized with violent pain in the epigastric region, extending, as she said, to her heart; this was accompanied with great difficulty of breathing, nausea, and a sinking sensation in the stomach. The bowels were very costive, having a motion about once in ten or twelve days, which was clay coloured. There was no appetite; and any solid or fluid taken into the stomach was immediately rejected. In this state she continued six or seven weeks, and was then seized with purgings and violent pain throughout the abdominal cavity; during this time, her sufferings were increased by the piles, causing a constant tenesmus; the purging motions were light coloured, and very offensive. Thus she alternately went on until six weeks before her death, when she was attacked with violent and acute pain about the right hypochondrium, extending to the right iliac region, and remaining fixed several days; the sickness was distressing to the greatest degree, nothing could be retained in the stomach; the bowels were firmly locked up, and the countenance of an intense yellow colour. Glysters were administered, without procuring the desired relief, and the abdomen was acutely painful to the touch, especially about the iliac region. Her sufferings were prolonged between five and six weeks, with only a day or two intermission of pain, until death happily released her.

I regretted much not having witnessed this case from the commencement of the disease; the above symptoms I collected from an intelligent nurse, who had accurately watched her throughout the complaint. The nature of the case could not be mistaken, and had I not been forcibly struck with the pain about the iliac region, I should not have entered so minutely into it. Here, I was induced to believe, some inflammatory action had gone on independently of the hepatic disease, together with the extreme torpid state of the bowels, as she had not been relieved by stool for nearly five weeks. I was anxious to obtain permission to examine the body, which was instantly granted me. About six hours after death, I proceeded with my examinations.

Externally, the whole body was extremely emaciated, the skin of an intense yellow colour, and the abdomen much distended with flatus. As soon as the abdominal cavity was exposed, the intestines protruded, of a dark purple hue, and exceedingly distended with flatus, having several patches of coagulable lymph interspersed upon their surface. Before I could proceed with the dissection, I gave vent to the air in the intestines, which had become distended considerably beyond their natural diameter. The omentum very vascular and thickened, firmly adhered to the whole surface of the stomach, nor could it be separated without rupturing the external surface of the stomach; it also adhered to the inferior edge of the liver and fundus of the gall bladder. The internal surface of the stomach was thickened and vascular, and the contents very offensive, being of a dark brown colour. The liver had undergone considerable disease; it was smaller than natural, hard, and its inferior edge completely schirrous, puckered, and adhering firmly to the gall bladder throughout its whole extent. The internal structure was tuberculated and white. The gall bladder was distended with very viscid bile, and its coats thickened by several layers of coagulable lymph. The ductus communis choledochus greatly distended, thickened, and containing a calculus weighing four drachms and ten grains. Many calculi were seen formed in the bile, which were soft, and yielded to the pressure of the fingers. Tracing the course of the intestines, my attention was attracted by a firm adhesion of the upper part of the ilium to the internal iliac muscle, by means of a tendinous-like band, which completely obliterated its canal; this continued nearly three inches. Having carefully dissected it out, I made a drawing, shewing a portion of the ilium above the stricture and mesentery, very much thickened, and a part of the obliterated canal. The mesentery was extremely vascular, and its glands astonishingly enlarged, and one or two indurated. The cæcum was healthy, colon and rectum natural, but contained no flatus. The kidnies and pelvic viscera were free from disease.

References to the Drawing.

. The tendinous band, obliterating the canal nearly three inches.
2. The upper portion of the ilium.
3. The intestine emerging from the band.
4 The mesentery, thickened and vascular.

H. W. BAILEY.

*Thetford, December* 3, 1815.

## Figures and Tables

**Figure f1:**